# Genomic Regions Associated With Gestation Length Detected Using Whole-Genome Sequence Data Differ Between Dairy and Beef Cattle

**DOI:** 10.3389/fgene.2019.01068

**Published:** 2019-11-05

**Authors:** Deirdre C. Purfield, Ross D. Evans, Tara R. Carthy, Donagh P. Berry

**Affiliations:** ^1^Animal & Grassland Research and Innovation Centre, Teagasc, Cork, Ireland; ^2^Irish Cattle Breeding Federation, Cork, Ireland

**Keywords:** gestation, sequence, single nucleotide polymorphism, genome-wide association, mixed model, bioinformatics, pregnancy

## Abstract

While many association studies exist that have attempted to relate genomic markers to phenotypic performance in cattle, very few have considered gestation length as a phenotype, and of those that did, none used whole genome sequence data from multiple breeds. The objective of the present study was therefore to relate imputed whole genome sequence data to estimated breeding values for gestation length using 22,566 sires (representing 2,262,706 progeny) of multiple breeds [Angus (AA), Charolais (CH), Holstein-Friesian (HF), and Limousin (LM)]. The associations were undertaken within breed using linear mixed models that accounted for genomic relatedness among sires; a separate association analysis was undertaken with all breeds analysed together but with breed included as a fixed effect in the model. Furthermore, the genome was divided into 500 kb segments and whether or not segments harboured a single nucleotide polymorphism (SNP) with a P ≤ 1 × 10^-4^ common to different combinations of breeds was determined. Putative quantitative trait loci (QTL) regions associated with gestation length were detected in all breeds; significant associations with gestation length were only detected in the HF population and in the across-breed analysis of all 22,566 sires. Twenty-five SNPs were significantly associated (P ≤ 5 × 10^-8^) with gestation length in the HF population. Of the 25 significant SNPs, 18 were located within three QTLs on Bos taurus autosome number (BTA) 18, six were in two QTL on BTA19, and one was located within a QTL on BTA7. The strongest association was rs381577268, a downstream variant of *ZNF613* located within a QTL spanning from 58.06 to 58.19 Mb on BTA18; it accounted for 1.37% of the genetic variance in gestation length. Overall there were 11 HF animals within the edited dataset that were homozygous for the T allele at rs381577268 and these had a 3.3 day longer (P < 0.0001) estimated breeding value (EBV) for gestation length than the heterozygous animals and a 4.7 day longer (P < 0.0001) EBV for gestation length than the homozygous CC animals. The majority of the 500 kb windows harboring a SNP with a P ≤ 1 × 10^-4^ were unique to a single breed and no window was shared among all four breeds for gestation length, suggesting any QTLs identified are breed-specific associations.

## Introduction

Optimal reproductive performance is a key contributor to profit in both dairy (Shalloo et al., 2014) and beef (Diskin and Kenny, 2016) cattle production systems. Calving interval, defined as the number of days between successive calving events in a cow, is often used as a metric of reproductive performance in cattle (Roche et al., 2017). Calving interval, however, is a complex phenotype influenced by the ability of the cow to 1) return to cyclicity early post-calving, 2) express (intense) estrus, 3) conceive, 4) establish and maintain pregnancy, and 5) gestation length. Heritable genetic variability in all five components of calving interval has been documented in dairy and beef cattle (Hansen et al., 2004; Berry et al., 2014). Nonetheless, traditional reproductive traits in cattle are generally lowly heritable implying that only a small proportion of the phenotypic variability is explainable by measurable additive genetic variation estimated from the various statistical models applied (Berry and Evans, 2014). Gestation length, however, is in fact moderately heritable, with direct heritability estimates in cattle documented to range from 0.33 to 0.62 (Hansen et al., 2004; Mujibi and Crews, 2009; Norman et al., 2009); gestation length has also been reported to be moderately heritable in humans (0.25 to 0.32; Clausson et al., 2000) and other species (0.30 in pigs – Rydhmer et al., 2008; 0.25 to 0.26 in sheep – Babar, 2008).

The moderate heritability of gestation length implies that a relatively accurate prediction of the ensuing phenotype could be realized, should a considerable proportion of the additive genetic variability in gestation length be predicted. Because of the relatively random segregation of homologous chromosomes during gametogenesis, it is not possible to achieve a high accuracy of prediction of genetic merit based on pedigree information alone. Hence, predictions based on genotype should yield more precise estimates of genetic merit. Moreover, predictions of genetic merit for a whole gamut of traits tend to be based on genomic merit estimated from the entire genome (Hayes et al., 2009). This hinders the optimal design of both breeding and mating schemes to accelerate the rate of gain in gestation length through the mating of complementary parents which achieve short gestation length through divergent biological processes (i.e., underlying genes and pathways). There is a paucity of studies, in cattle at least (Maltecca et al., 2011; Fang et al., 2019), that have attempted to elucidate the genomic features of gestation length; to our knowledge, no such study has been undertaken using whole genome sequence data in multiple breeds.

The objective therefore of the present study was to attempt to locate genomic regions contributing to differences in gestation length. This was achieved using a population of 22,566 sires from four different breeds each with gestation length breeding values derived from the phenotypes of multiples more of their descendants.

## Methods

The data used in the present study originated from a pre-existing database managed by the Irish Cattle Breeding Federation (ICBF). Therefore, it was not necessary to obtain animal care and use committee approval in advance of conducting this study.

### Phenotypes

Estimated breeding values (EBVs) for direct gestation length and their associated reliabilities were obtained from the ICBF database from the December 2018 national genetic evaluation. All estimates were obtained from a univariate multi-breed model where the heritability of direct gestation length was assumed to be 0.38. Gestation length phenotypes from 4,735,484 births were used in the genetic evaluation with a pedigree containing 25,469,990 animals. Of the animals with generated EBVs, only purebred (i.e., ≥87.5% of a single breed) genotyped sires of any of four breeds were retained for analysis; Angus (AA), Charolais (CH), Holstein-Friesian (HF), and Limousin (LM). The effective record contribution (ERC) of each sire, taking into consideration genotyped animals, was estimated using the Harris and Johnston method (Harris and Johnson, 1998); only animals with an ERC ≥1 were retained for use in the present study. Deregression of the EBVs was completed using the secant method with a full animal model pedigree file in the MiX99 software suite (Strandén and Mäntysaari, 2010). After edits, 22,566 sires from four breeds were available for analysis which included 2,308 AA, 2,327 CH, 14,759 HF and 3,172 LM. The median ERC within each breed was 1.88, 1.22, 1.11, and 1.22 within the AA, CH, HF and LM populations, respectively whereas the mean EBV reliability was 63.74%, 60.13%, 58.79% and 59.80%, respectively. The median ERC across all breeds was 1.16 and the mean EBV reliability was 59.58%. The mean EBV and SD per breed for all 22,566 sires are detailed in **Table S1**, as well as the average phenotypic gestation length and SD for all purebred females within each of the four breeds.

### Genotype Data

All genotypes of the 22,566 sires with gestation length EBVs were imputed to whole genome sequence as part of a larger dataset of 638,662 genotyped animals from multiple breeds as detailed previously by Purfield et al. (2019). All animals included in the present study were genotyped on a variety of genotyping panels including the Illumina High Density [HD; n = 1,845; 777,962 single nucleotide polymorphism (SNPs)], Illumina Bovine SNP50 (n = 7,389; 54,001 SNPs), or the custom Irish Dairy and Beef (IDB) V1 (n = 7,608; 16,622 SNPs), IDBV2 (n = 4,994; 16,223 SNPs), or IDBV3 (n = 730; 52,445 SNPs) genotype panels. All 638,662 genotyped animals had a call rate ≥90% and only autosomal SNPs, SNPs with a known chromosome and position on UMD 3.1, and only SNPs with a call rate ≥90% were retained within each panel.

Prior to imputation to whole genome sequence (WGS), all genotyped animals in the larger 638,662 genotyped dataset were first imputed to HD using a two-step approach in FImpute2 (Sargolzaei et al., 2014); this involved imputing all IDB-genotyped animals to the Bovine SNP50 density and subsequently imputing all resulting genotypes, including the Bovine SNP50 genotypes, to HD using a multi-breed reference population of 5,504 HD genotyped animals. Imputation of all 638,662 HD imputed animals to WGS was then undertaken using a reference population of 2,333 *Bos Taurus* animals of multiple breeds from Run6.0 of the 1000 Bulls Genomes Project. Imputation of the HD genotypes to WGS was achieved by firstly phasing all 638,662 HD imputed animals using Eagle (Loh et al., 2016; version 2.3.2) and subsequently imputing all animals to WGS using minimac3 (Das et al., 2016).

To further refine the WGS imputed dataset consisting of 22,566 sires with genotype and EBV information in the present study, all SNPs with a minor allele frequency (MAF) <0.005 across all animals were removed for the multi-breed analysis; additionally SNPs with a minor allele frequency (MAF) <0.005 within each breed were removed for the respective within-breed analysis. Furthermore, regions with possible poor WGS imputation accuracy (n = 687,137 SNPs) were identified using a dataset of 147,309 verified parent-progeny relationships from the 638,662 genotyped dataset as previously described by Purfield et al. (2019). Following edits, 15,878,111 imputed SNPs remained for analysis across all breeds while 14,727,399, 16,100,467, 14,248,828 and 16,140,453 imputed SNPs remained for the AA, CH, HF, and LM breeds, respectively.

### Genome-Wide Association Analyses

Whole genome association analyses were performed within each breed separately, as well as in a dataset of all breeds combined, using an animal linear mixed model in Wombat (Meyer and Tier, 2012). The VanRaden Method I (VanRaden, 2008) was used to estimate a genomic relationship matrix based on just the imputed autosomal SNPs from the edited HD panel (n = 642,153 SNPs). All imputed sequence SNPs, scored as 0, 1 or 2, were included individually as a fixed effect covariate in the model one at a time. Breed was included as a fixed class effect for the multi-breed analyses. Each dependent variable was also weighted using the approach outlined by Garrick et al. (2009);

$$w_{i}=\;\frac{1-h^{2}}{\left[c+\frac{1-r_{i}^{2}}{r_{i}^{2}}\right]h^{2}}$$

where *w_i_ w_i_* is the weighting factor of the *i*th deregressed EBV, *h^2^ h^2^* is the heritability estimate for the trait in question, is the reliability of the *i*th deregressed EBV and c is the proportion of genetic variance not accounted by the SNPs and set at 0.9 for analyses thus allowing each SNP to attribute up to 10% of the genetic variance. Test statistics for all SNPs were obtained and converted into their corresponding p-values. The genomic inflation factor was estimated within and across all breeds and ranged from 0.98 in the CH population to 1.02 in the LM population; the genomic inflation factor in the across-breed analysis was 0.999. A genome-wide SNP significance threshold of P ≤ 5 × 10^-8^ and a suggestive threshold of P ≤ 1 × 10^-5^ were applied to the results from each analysis.

The proportion of the genetic variance attributable to individual SNPs was calculated as 2*pqa*^2^/*σ^2^*, where *p* was the major allele frequency, *q* was the minor allele frequency, *a* represented the estimated allele substitution effect, and *σ^2^* was the genetic variance for gestation length.

### Defining Quantitative Trait Loci

All SNPs above the suggestive threshold (P ≤ 1 × 10^-5^) were used for defining quantitative trait loci (QTL) regions associated with gestation length. To estimate the QTL start and end positions, all SNPs within a 5 Mb window and in strong linkage disequilibrium (LD) (r^2^ of ≥0.7) with each suggestively associated SNP were considered to be part of the QTL. Overlapping QTL were merged together and considered as the same QTL. To limit the number of false positive QTLs, a minimum of two suggestively associated SNPs had to be present in the QTL region. Genes within and overlapping each QTL were identified using NCBI map viewer (http://www.ncbi.nlm.nih.gov/mapview) and Ensembl (http://ensemble.org) based on the bovine UMD 3.1 build. Candidate genes were chosen from QTL based on previous literature and their biological function. If no gene resided in the QTL region, genes within 250 kb of the start and end position of the QTL, were considered as putative candidate genes. Previously reported cattle QTL were obtained from the animal QTLdb (http://www.animalgenome.org/cgi-bin/QTLdb/index). In addition, to identify QTL present in more than one breed, each chromosome was split into 500 kb windows and each window that contained a SNP with a P ≤ 1 × 10^-4^ present in two or more breeds, was considered a putative across-breed QTL. This threshold was previously applied by Tenghe et al. (2016) when detecting across-trait QTLs thus enabling the identification of putative across-breed genomic regions with less stringency.

### SNP Effect Directions

To determine if significant SNPs are currently lengthening or shortening gestation length at a population level, the direction of the allele substitution effect for the major allele for all SNPs with a P ≤ 1 × 10^-4^ was determined in each breed. In addition, the proportion of these SNPs within each breed that differed in their allele effect direction when compared to each of the other breeds was also determined. This was then categorized according to the SNP effect size, in that small represented SNPs with an allele effect size between -0.8 and 0.8 days, medium represented SNPs with an allele effect size between -0.8 and -1.58 days or between 0.8 to 1.58 days, and large represented SNPs with an allele effect size of >|1.58| days. On average per breed, >|1.58| days represents the top 5% of SNPs with the largest effect size, -0.8 and -1.58 days or between 0.8 to 1.58 days represents, on average, the top 25 to 5% of SNP effect sizes and small is the remaining 75% SNP effect sizes. The SNP effect size did not have to be in the same effect size category in the breed comparison - a SNP with a large allele substitution effect in the AA population may have had a small allele substitution effect in the CH population but rather it was the SNP allele effect direction that was of interest.

### Pathway Analysis

All genes located within QTLs, or within 250 kb of the start/end of QTLs where no genes were identified, were used to identify over-represented gene ontology (GO) terms and pathways associated with gestation length within each breed and across all breeds using the Database for Annotation, Visualization and Integrated Discovery (DAVID) v.6.8. All associated P-values were calculated by EASE (an adoption of the Fisher Exact test to measure the gene-enrichment in annotation terms) and Benjamini-Hochberg was used to correct for multiple testing.

## Results

Putative QTL regions associated with gestation length were detected in all breeds. Despite the moderate population sizes of the individual breeds in the present study, significant associations with gestation length were only detected in the HF population and in the across-breed analysis of all 22,566 sires. In total the 22,566 sires represented 2,262,706 progeny with gestation length records which were included in the genetic evaluation.

### Angus

**Figure 1:**
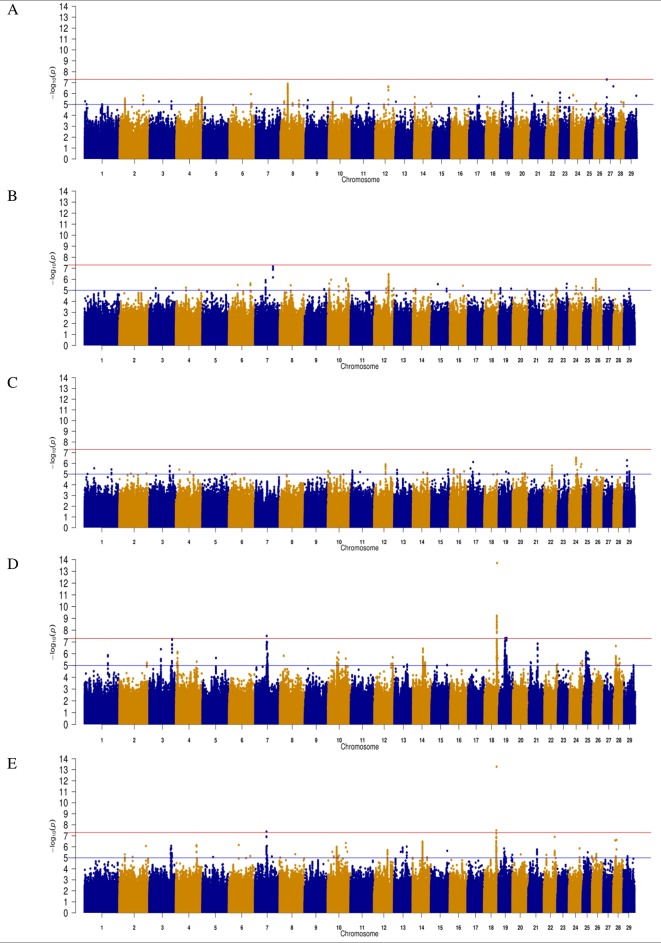
Manhattan plots for direct gestation length in **(A)** Angus **(B)** Charolais **(C)** Limousin **(D)** Holstein-Friesian and **(E)** across all breeds. The red line indicates the genome wide significance threshold of P ≤ 5 × 10^-8^ and the blue is the suggestive significance threshold of P ≤ 1 × 10^-5^.

A total of 311 SNPs were suggestively associated (P ≤ 1 × 10^-5^) with direct gestation length in the AA population (**Figure 1**) and consequently 25 QTLs in 14 different Bos taurus autosome numbers (BTAs) were suggested (**Table 1**). No SNP was above the significance threshold, although one QTL on BTA27 from 6.71 to 6.75 Mb contained SNPs that were tending towards significance (p = 5.13 × 10^-8^). Of the 9 suggestively associated SNPs within this QTL on BTA27, 6 were intronic variants within the *ASB5* gene and the strongest association accounted for 5.35% of the genetic variation in gestation length. Eight of the 9 suggestively associated SNPs were in complete LD (r^2^ = 1) within the AA population. All 9 SNPs were segregating at a greater frequency in the CH, LM and HF breeds (MAF ranged 0.05 to 0.19) than in the AA population, but they were only marginally significant within the HF population (P-value ranged from 0.03 to 0.05). The QTL containing the greatest number of suggestively-associated SNPs with gestation length in the AA population was located on BTA8 spanning 32.80 to 33.69 Mb; 53 intergenic SNPs were suggestively associated. No plausible candidate gene was identified within or 250 kb up or downstream of this QTL but two spliceosomal RNAs were located within the QTL (*ENSBTAG00000028923* and *ENSBTAG00000043919*). The pattern of significance level by SNP within this QTL followed a bell curve shape suggesting that this was a true association rather than imputation error. The QTL from 24.94 to 25.06 Mb on BTA2 yielded 12 suggestively-associated SNPs which varied in LD from r^2^ 0.75 to 1. The strongest variant within this QTL on BTA2, rs382826516, explained 4.41% of the genetic variance in gestation length. Two plausible candidate genes were located within this QTL (i.e., *CYBRD1* and *DCAF1*) and all 12 SNPs were segregating to a similar extent in the other three breeds. Similar to the strongest QTL association on BTA27, the 12 SNPs within this QTL on BTA2 were only marginally significant in the HF population (P-value ranged from 0.02 to 0.05) but were not significant in either the CH or LM populations. A large number of suggestively associated variants (n = 41) were also detected on BTA24 in a QTL located between 26.56 and 26.79 Mb; all associated SNPs within this QTL were intergenic variants in complete LD (r^2^ = 1), with an individual SNP MAF of 0.019 and no gene was identified in the QTL region. The frequency of all 41 variants was also low in both the CH and LM populations with the allele frequency associated with longer gestation length ranging from 0.015 to 0.023 and from 0.022 to 0.026, respectively in the two populations. Indeed no homozygous reference allele carriers for any of the 41 SNPs were identified in any of the four breeds examined in the present study. When the boundary of this QTL on BTA24 was extended by 250 kb up and downstream in an attempt to identify candidate genes, 3 genes were identified (*DSC1, DSC2, DSC3*) of which *DSC3* was in closest proximity (111 kb upstream). Although no suggestively-associated SNPs were identified in *DSC3* itself, animals containing all 41 heterozygous SNPs did have a longer (P < 0.001) gestation length EBV than those that did not (heterozygous mean EBV 0.51, S.D 3.19, n = 89; homozygous alternate allele -1.08, S.D 3.05, n = 2,219). The only variant class enriched for association within the AA population was the intergenic SNP class (n = 246) with a fold enrichment of 1.17 in comparison to what would be expected by chance.

**Table 1:** Quantitative trait loci (QTL) associated with gestation length in the Angus population*.

BTA	Start	End	No SNP <10^-5^	Strongest SNP	Strongest SNP position	+ Allele	+ Allele Freq	P-value	No genes	Candidate gene(s)
2	24937082	25055889	12	rs382826516^a^	25000379	A	0.274	2.91x10^-6^	1	CYBRD1, DCAF1
2	107904357	107907047	3	rs135564449^b^	107904357	C	0.534	1.50x10^-6^	1	ATG9A^†^, GLB1L
4	100539852	100625859	19	rs133278564^c^	100562856	C	0.250	8.89x10^-6^	1	MTPN^†^
4	114304399	114326048	3	rs382841510^a^	114304628	T	0.166	3.36x10^-6^	1	KCNH2
4	116394356	116460525	20	rs136426377^a^	116457230	G	0.578	2.24x10^-6^	0	
6	97978568	98182029	2	rs110371749^a^	97978568	C	0.009	1.14x10^-6^	1	RASGEF1B
8	16928207	16928585	2	rs459357437^c^	16928585	T	0.992	5.27x10^-6^	1	MOB3B^†^
8	32808372	33685759	53	rs480101726^a^	33057972	T	0.040	1.28x10^-7^	0	ENSBTAG00000028923, ENSBTAG00000043919
8	84301275	84314848	3	rs137312367^a^	84310591	G	0.208	4.37x10^-6^	0	
10	18291156	18319748	2	rs465012804^a^	18305547	C	0.802	6.08x10^-6^	0	
10	102186156	102247635	21	rs379454827^c^	102231543	A	0.008	2.40x10^-6^	1	ENSBTAG00000046684^†^
11	77745444	77790835	2	rs381959206^a^	77788466	C	0.005	8.89x10^-6^	0	
12	60057813	60148456	16	rs209718350^a^	60064563	G	0.951	2.27x10^-6^	0	
14	4506937	4582353	5	rs385012447^c^	4551331	A	0.009	2.08x10^-6^	1	TRAPPC9^†^
19	9163293	9243910	7	rs210497903^a^	9234427	A	0.159	8.92x10^-6^	2	ENSBTAG00000035001,ENSBTAG00000037424
19	58003494	58022541	2	rs41928801^a^	58003628	A	0.098	1.29x10^-6^	0	
19	58737200	58750837	4	rs380825275^a^	58742358	G	0.011	9.20x10^-7^	0	
19	58764123	58907205	6	rs468076774^a^	58764123	T	0.009	1.20x10^-6^	1	SLC39A11
21	21703977	22313839	2	rs442241598^a^	21881137	T	0.038	9.61x10^-6^	17	
23	3769904	3863939	24	rs436186743^a^	3782972	A	0.008	8.29x10^-7^	0	
24	11009555	11020853	17	rs137177798^a^	11016075	A	0.614	1.35x10^-6^	0	
24	26564594	26787383	41	rs111002801^a^	26670279	C	0.019	4.94x10^-6^	0	DSC3
27	6710713	6756338	9	rs210917556^c^	6731141	T	0.019	5.13 x10^-8^	2	ASB5^†^,SPCS3
27	36434276	36445229	2	rs385393759^a^	36434276	G	0.008	2.19 x10^-7^	0	
28	37301163	37315868	10	rs42150599^a^	37301163	G	0.173	6.54x10^-6^	0	

*BTA, Bos taurus autosome number; SNP, name of single nucleotide polymorphism. No SNP <10^-5^ is the number of SNPs within the QTL with a p-value ≤ 1 × 10^-5^. P-value is the p-value of strongest SNP association. + Allele is the allele that had a positive SNP effect on gestation length. No of genes is the number of genes in the QTL. If no gene was present in the QTL the nearest functional candidate gene within 250 kb was chosen. ^a^intergenic, ^b^upstream gene variant, ^c^intronic, ^†^gene in which the most significant SNP within the QTL was identified in.

### Charolais

Similar to the AA population, no significant associations for direct gestation length were identified in the CH population, although 189 SNP were suggestively associated (**Figure 1**). Seventeen QTLs on 11 different BTAs were identified (**Table 2**). The strongest association, rs378335003 (P = 6.90 × 10^-8^), was located in a QTL spanning from 82.27 to 82.29 Mb on BTA7 along with 7 other suggestively-associated intergenic SNPs. No gene was located within the QTL boundary but the candidate gene *TENM2* was located just 90 kb downstream. Although variants were segregating within *TENM2*, none were significant, suggesting this QTL may play a regulatory role in the expression of *TENM2*. The second strongest QTL association was on BTA12 from 66.46 to 66.56 Mb where 41 suggestively-associated SNPs resided. All 41 SNPs were intronic variants located within the *GPC5* gene, although the alleles conferring longer gestation were near fixation as the frequency of all 41 variants ranged from 0.895 to 0.994 in the CH population. Similar allele frequencies were detected in the other two beef breeds evaluated (i.e., Angus and Limousin) but failed to associate with gestation length suggesting that this is a possible breed-specific association. A similar observation was also observed for the QTL on BTA26 in that alleles were segregating at similar frequencies in all three beef breeds evaluated in the present study but 13 intronic variants located within the *SORBS1* gene only achieved suggestive significance in the CH population. Intronic variants were the only SNP class identified to be enriched for association within the CH population with a fold enrichment of 1.48.

**Table 2:** Quantitative trait loci (QTL) associated with gestation length in the Charolais population.

BTA	Start	End	No SNP <10^-5^	Strongest SNP	Strongest SNP position	+ Allele	+ Allele Freq	P-value	No genes	Candidate gene(s)
6	98666318	98859289	8	rs449564357^a^	98839959	G	0.990	2.37x10^-6^	0	
7	48561938	48632722	16	rs137784361^a^	48584512	T	0.994	1.18x10^-6^	1	Neorog1
7	82270231	82292203	8	rs378335003^a^	82291415	C	0.989	6.90x10^-8^	0	TENM2
9	90109473	90115186	3	rs43606567^c^	90115186	T	0.448	7.78x10^-6^	1	ESR1^†^
10	7291137	7350492	10	rs109163850^a^	7318841	A	0.930	2.44x10^-6^	1	SV2C
10	7659329	7679958	3	rs109797643^c^	7673663	T	0.496	6.77 x10^-6^	1	IQGAP2^†^
10	83696836	83718598	3	rs43645033^a^	83709850	T	0.562	8.80x10^-7^	0	
10	93535961	93585084	3	rs43649425^b^	93571828	T	0.086	2.72x10^-6^	1	TSHR^†^
10	95050679	95101064	19	rs42977185^a^	95068244	G	0.696	4.48x10^-6^	0	
12	66043799	66213706	20	rs383596163^a^	66259397	C	0.993	4.29x10^-7^	0	
12	66457354	66655558	41	rs386112721^c^	66539912	T	0.993	3.49x10^-7^	1	GPC5^†^
14	13834084	13920775	3	rs135227220^a^	13899228	C	0.986	7.99x10^-6^	0	
18	32926687	32951737	2	rs383154371^a^	32926687	T	0.956	9.97x10^-6^	0	
19	8627909	8640553	3	rs207813952^a^	8630179	G	0.334	6.35x10^-6^	0	
22	54920078	54978460	6	rs443749810^c^	54961292	A	0.976	7.13x10^-6^	2	SEC13^†^ GHRL
24	32655123	32659852	2	rs381975298^a^	32657250	T	0.973	4.39x10^-6^	0	
26	16865291	16924272	13	rs211666967^c^	16869415	A	0.112	9.40x10^-7^	1	SORBS1^†^

*BTA, Bos taurus autosome number; SNP, name of single nucleotide polymorphism. No SNP <10^-5^ is the number of SNPs within the QTL with a p-value ≤ 1 × 10^-5^. P-value is the p-value of strongest SNP association. + Allele is the allele that had a positive SNP effect on gestation length. No of genes is the number of genes in the QTL. If no gene was present in the QTL the nearest functional candidate gene within 250 kb was chosen. ^a^intergenic, ^b^downstream gene variant, ^c^intronic, ^†^gene in which the most significant SNP within the QTL was identified in.

### Limousin

The LM population had the fewest suggestive associations of all breeds in the present study; only 179 suggestive associations were identified and these could be collapsed into 18 QTL across 13 BTAs (**Table 3**). The majority of these associations (44.13%) were located within a single QTL extending from 78.10 to 78.17 Mb on BTA15 and all were intronic variants in *C11orf49*. The frequency of the allele associated with longer gestations within this QTL was almost at fixation within the LM population with frequencies ranging from 0.992 to 0.995; similar frequencies existed in all other breeds with the exception of AA where the frequency ranged from 0.895 to 0.935. The strongest association was the intergenic variant rs382939180 within the QTL spanning from 32.46 to 32.47 Mb on BTA24 but it only explained 3.30% of the genetic variance in gestation length. No gene was identified within this QTL although 3 genes were located within 250 kb downstream including *HRH4*, *IMPACT* and *OSBPL1A*. Interestingly the missense variant rs210280020 within *MYCBP2* was the strongest association observed within the QTL on BTA12, although the disruptive C allele was predicted to be tolerated [Sorting Intolerant from Tolerant (SIFT) score of 0.61]. As 102 of the 179 suggestive associations were intronic variants, this SNP class was substantially enriched for associated variants (fold enrichment 2.19).

**Table 3:** Quantitative trait loci (QTL) associated with gestation length in the Limousin population*.

BTA	Start	End	No SNP <10^-5^	Strongest SNP	Strongest SNP position	+ Allele	+ Allele Freq	P-value	No genes	Candidate gene(s)
1	122986997	123110634	3	rs447613174^a^	123066190	G	0.993	3.66 x10^-6^	1	PLSCR1
3	93753165	93935370	4	rs439859698^a^	93785630	C	0.979	1.74 x10^-6^	2	PODN,SCP2
10	2496163	2604788	5	rs454662674^a^	2507549	C	0.977	5.10 x10^-6^	0	
11	8496063	8536679	2	rs135066905^a^	8517491	T	0.620	4.71 x10^-6^	0	
12	52544063	52635583	8	rs210280020^d^	52606206	T	0.968	1.93 x10^-6^	1	MYCBP2^†^
13	14035366	14356554	4	rs378665620^a^	14040023	T	0.993	4.06 x10^-6^	0	
14	49080514	49117448	3	rs381835043^a^	49080514	C	0.994	7.20 x10^-6^	0	
14	67771168	67873947	7	rs377884869^c^	67860285	T	0.114	8.00 x10^-6^	1	STK3^†^
15	78103186	78175580	79	rs379711605^c^	78144110	G	0.993	3.79 x10^-6^	1	C11orf49^†^
16	18332592	18395061	8	rs136829849^a^	18395061	A	0.741	3.57 x10^-6^	1	SNORA41
17	52403981	52418867	2	^a^	52403981	T	0.993	9.62 x10^-6^	0	
22	36379583	36379663	2	^a^	36379661	C	0.013	7.83 x10^-6^	0	
22	36460279	36460289	2	rs466503122^a^	36460289	C	0.014	1.62 x10^-6^	0	
22	36467895	36467913	2	rs111742368^a^	36467913	G	0.015	3.24 x10^-6^	0	
24	32458959	32472833	5	rs382939180^a^	32466786	G	0.809	2.91 x10^-7^	0	HRH4, IMPACT,OSBPLIA
24	37737096	37959307	4	rs208627686^b^	37834560	G	0.524	4.04 x10^-6^	4	MYL12B^†^
29	16015868	16099153	6	rs210179107^a^	16043942	C	0.872	5.13 x10^-6^	0	
29	26742643	26788237	4	rs136687125^b^	26755302	C	0.720	5.68 x10^-6^	2	ENSBTAG00000010433^†^

*BTA, Bos taurus autosome number; SNP, name of single nucleotide polymorphism. No SNP <10^-5^ is the number of SNPs within the QTL with a p-value ≤ 1 × 10^-5^. P-value is the p-value of strongest SNP association. + Allele is the allele that had a positive SNP effect on gestation length. No of genes is the number of genes in the QTL. If no gene was present in the QTL the nearest functional candidate gene within 250 kb was chosen. ^a^intergenic, ^b^upstream gene variant, ^c^intronic, ^d^missense, ^†^gene in which the most significant SNP within the QTL was identified in.

### Holstein-Friesian

In contrast to the analyses of the three beef breeds, 25 SNPs were significantly associated (P ≤ 5 × 10^-8^) with direct gestation length in the HF population and a further 696 SNPs surpassed the suggestive threshold. Of the 25 significant SNPs, 18 were located within three QTLs on BTA18, 6 were in two QTL on BTA19 and one was located within a QTL on BTA7 (**Table 3**). In total, 28 distinct QTL across 15 BTAs were associated with gestation length in the HF population. The strongest association was rs381577268, a downstream variant of *ZNF613* located within a QTL spanning from 58.06 to 58.19 Mb on BTA18 and it accounted for 1.37% of the genetic variance in gestation length. Overall, there were 11 HF animals within the edited dataset that were homozygous for the T allele at rs381577268 and these had a 3.3 day longer (P < 0.001) EBV for gestation length than the heterozygous animals and a 4.7 day longer (P < 0.001) EBV for gestation length than the homozygous CC animals. This was also reflected in their corresponding calving difficulty and mortality breeding values where homozygous TT animals had a greater (P < 0.001) mean EBV for direct calving difficulty (mean EBV 0.12 units; s.d. 0.12; n = 11) and mortality (P < 0.05) (mean EBV -0.015 units; s.d 0.01; n = 11) than homozygous CC animals (mean calving difficulty EBV-0.062, s.d 0.06, n = 14,170; mean mortality EBV -0.008, s.d 0.01, n = 14,170). The pedigree of all 11 homozygous TT sires was traced back and examined for 6 generations where possible, but no common ancestor was detected. The allele frequency of the T allele (i.e., associated with longer gestation length) was low within the HF population with a frequency of 0.020 but similar to observed in the beef populations where it was only marginally segregating (0.007) in the AA population and below the MAF threshold for inclusion in the analysis in both the CH and LM populations (i.e., MAF <0.005). Although the MAF of rs381577268 was below threshold in the CH and LM populations, it was included in a post-hoc analysis to test if it was significantly associated with gestation length within these breeds; it was found to be non-significant with p-values of 0.319 and 0.335, respectively. Similarly, rs381577268 was also non-significant in the AA population (p = 0.16). Indeed, no SNP within the QTL region extending from 58.06 to 58.19 on BTA18 was suggestively associated (P ≤ 1 × 10^-5^) with gestation length in any of the beef populations but several SNP located <40 kb upstream of *ZNF613* had a P between 0.001 and 0.05 within the CH and LM populations.

A total of four missense variants were also identified to be suggestively associated with gestation length in the HF population; 2 were located within the *ABCA13* gene in a QTL on BTA4 situated between 7.04 to 7.52 Mb whereas the remaining two SNPs were located in two separate QTL on BTA18. Of these four missense variants, only one, rs483267294 (P = 2.32 × 10^-6^) located in *ENSBTAG00000039212* and within the QTL from 57.48 to 57.59 Mb on BTA18, was predicted to be deleterious (SIFT 0.03). Although the A allele was predicted to be deleterious, the frequency of this allele was relatively low in the HF population (0.051) and ranged from 0.018 to 0.085 in the beef populations. The LD (r^2^) between this missense variant rs483267294 and the downstream variant rs381577268 of *ZNF613* within the HF population was just 0.31 suggesting that it was not tagging the same association but rather two possible separate QTLs associated with gestation length. Indeed no suggestively associated SNP in the QTL on BTA18 from 57.48 to 57.59 was in strong LD (r^2^ < 0.4) with any of the SNPs in the QTL from 58.06 to 58.19 Mb. Six genes were located within the QTL from 57.48 to 57.59 Mb of which the upstream variant of *KLK14*, rs384897226, exhibited the strongest association (P = 4.87 × 10^-7^) but it only explained 0.66% of the genetic variance in gestation length. A third QTL was also detected further upstream on BTA18 from 55.92 to 57.02 Mb, suggesting that the 3-Mb genomic region from 55.92 Mb to 58.19 Mb may harbor multiple genes associated with gestation length. In total, 17 SNPs were significant and a further 46 were suggestively associated with gestation length within this 3-Mb region.

Significant associations with gestation length were also detected on BTA7 and BTA19 (**Table 4**), the strongest of which was located on BTA7 (P = 3.06 × 10^-8^). Although 83 genes were located in the wide QTL on BTA7 (51.61 to 56.34 Mb), 11 of the 54 suggestively-associated SNPs were intronic variants within *CYSTM1* and the most significant variant explained 1.08% of the genetic variance. From the enrichment analyses, three SNP classes including upstream (fold enrichment 1.49), intronic (fold enrichment 1.17) and synonymous variants (fold enrichment 3.61) were enriched suggesting gene regulation contributes to direct gestation length within the HF population.

**Table 4:** Quantitative trait loci (QTL) associated with gestation length in the Holstein-Friesian population*.

BTA	Start	End	No SNP <10^-5^	Strongest SNP	Strongest SNP position	+ Allele	+ Allele Freq	P-value	No genes	Candidate gene(s)
1	106544365	106573019	6	rs211674422^a^	106562729	G	0.795	1.35 x10^-6^	0	
2	125797480	125824150	8	rs134618553^a^	125822265	A	0.540	5.81 x10^-6^	0	
3	53231290	53254980	12	rs11015107^a^	53235224	C	0.010	4.11 x10^-7^	0	
3	103958875	104046559	2	rs799224682^c^	104046559	C	0.982	1.86 x10^-7^	3	TMEM269^†^
4	7044130	7518266	43	rs472554192^a^	7514459	A	0.021	7.10 x10^-7^	2	ABCA13,7SK
7	51609116	56336142	54	rs378423353^c^	53088085	T	0.124	3.06x10^-8^	83	CYSTM1^†^
7	56661674	56918810	2	rs109970665^a^	56803371	T	0.202	7.40 x10^-6^	0	
7	57127004	57315768	23	rs43519218^a^	57167198	A	0.155	9.72 x10^-7^	3	YIPF5,KCTD16
10	42266891	43301260	21	rs380551392^a^	42361205	C	0.928	2.07 x10^-6^	16	
10	48984533	49023546	3	rs381355858^c^	48984533	A	0.056	7.70 x10^-7^	1	RORA^†^
12	84384983	84384988	2	^a^	84384988	A	0.979	2.00 x10^-6^	0	
13	59061885	59108749	2	rs135350894^d^	59108749	A	0.734	8.41 x10^-6^	2	PMEPA1,ZBP1^†^
14	43501239	48599522	31	rs439739810^c^	46819750	A	0.959	3.68 x10^-7^	26	FABP9^†^
14	56445115	56647342	86	rs110601974^a^	56596172	T	0.034	5.56 x10^-6^	0	
18	55926709	57016965	63	rs383639920^c^	56551941	G	0.017	5.91 x10^-10^	60	CPT1C^†^
18	57482287	57591440	43	rs384897226^b^	57503131	T	0.054	4.84 x10^-7^	6	KLK14^†^,ENSBTAG00000039212
18	58059492	58193281	10	rs381577268^d^	58141989	T	0.020	1.97 x10^-14^	7	ZNF613
19	25993220	26579664	19	rs109632828^a^	26480940	A	0.644	1.54 x10^-6^	1	PITPNM3
19	26999418	27517741	7	rs381172322^a^	27483350	G	0.395	4.67 x10^-7^	27	
19	28459429	28983381	65	rs380899775^b^	28529700	G	0.136	4.91 x10^-8^	15	PFAS, SLC25A35^†^,
19	32888891	38468608	46	rs41912302^b^	35264878	C	0.841	4.61 x10^-8^	80	RAI1^†^
21	40861557	41148186	32	rs208954540^a^	40907918	A	0.390	1.38 x10^-7^	1	ENSBTAG00000027863
24	59171495	59180428	2	rs210181427^a^	59179570	C	0.625	4.19 x10^-6^	0	
25	13457962	13626389	57	rs384885964^c^	13478288	A	0.088	8.20 x10^-7^	1	PARN^†^
25	22121076	22148689	3	rs109616718^a^	22148423	T	0.230	9.30 x10^-7^	1	PRKCB
28	9756961	9775184	40	rs208635521^a^	9774271	A	0.647	2.20 x10^-7^	0	
28	17248068	17249161	2	rs459816768^a^	17248473	T	0.937	8.52 x10^-7^	0	
28	25271166	25382045	4	rs42142414^c^	25294572	A	0.614	2.67 x10^-7^	3	STOX1^†^,DDX50

*BTA, Bos taurus autosome number; SNP, name of single nucleotide polymorphism. No SNP <10^-5^ is the number of SNPs within the QTL with a p-value ≤ 1 × 10^-5^. P-value is the p-value of strongest SNP association. + Allele is the allele that had a positive SNP effect on gestation length. No of genes is the number of genes in the QTL. If no gene was present in the QTL the nearest functional candidate gene within 250 kb was chosen. ^a^intergenic, ^b^upstream gene variant, ^c^intronic, ^d^downstream gene variant, ^†^gene in which the most significant SNP within the QTL was identified in.

### Across-Breed Analysis

The across-breed analyses involving all 22,566 sires with breed fitted as a fixed effect identified 341 suggestive SNPs and 3 significant SNPs to be associated with direct gestation length. These associations consequently identified 24 unique QTLs across 14 BTAs (**Table 5**). These results were largely reflective of the within-breed HF association results due to the larger sample size of this population in comparison to the beef breeds. The strongest SNP association was rs381577268 (P = 5.25 × 10^-14^), the downstream variant of *ZNF613* on BTA18 that was previously identified in the HF population, although as it was the only suggestively associated SNP within this QTL it was not identified as a putative across-breed QTL. Nonetheless, two QTLs further upstream of rs381577268 on BTA18 were identified, suggesting the 3-Mb region from 55.5 Mb to 58.5 Mb may harbor a putative across-breed QTL.

**Table 5:** Quantitative trait loci (QTL) associated with gestation length across all 22,566 sires with gestation length phenotypes*.

BTA	Start	End	No SNP <10^-5^	Strongest SNP	Strongest SNP position	+ Allele	+ Allele Freq	P-value	No genes	Candidate gene(s)
2	25703163	25764224	2	rs440124614^d^	25703163	C	0.977	4.91 x10^-6^	1	ERICH2^†^
3	98841884	98849329	29	rs209841235^a^	98847024	T	0.132	1.37 x10^-6^	0	
3	100766422	100777480	3	rs42581438^b^	100773079	C	0.550	8.16 x10^-7^	1	MAST2^†^
3	104046559	104266624	6	^a^	104266624	C	0.988	3.86 x10^-6^	7	CLDN19
4	94319933	94818120	3	rs479683550^a^	94818120	T	0.995	7.17 x10^-7^	10	UBE2H
4	95321764	95321892	8	rs473707947^a^	95321870	A	0.066	8.50 x10^-7^	0	
7	52922609	53089185	25	rs378423353^c^	53088085	T	0.126	4.16 x10^-8^	4	CYSTM1^†^
7	55759705	55833318	2	rs382485411^a^	55801809	A	0.074	5.83 x10^-6^	1	ARHGAP26
10	28673906	28888361	28	rs522302467^c^	28849088	C	0.015	7.46 x10^-6^	2	AVEN,RYR3^†^
10	41772174	41856602	66	rs210399646^a^	41814534	C	0.150	1.00 x10^-6^	0	
10	83626502	83634162	2	rs207683997^d^	83626502	A	0.898	4.48 x10^-7^	1	SIPA1L1^†^
12	61738949	62374951	6	^a^	62343825	C	0.990	2.01 x10^-6^	0	
13	40292785	40396496	8	rs209154825^c^	40362715	T	0.881	1.17 x10^-6^	1	RALGAPA2^†^
13	59061885	59065514	3	rs209578693^c^	59064151	T	0.793	9.64 x10^-7^	1	PMEPA1^†^
14	44492347	48599522	30	rs210147540^a^	46786108	T	0.968	3.36 x10^-7^	20	PAG1
18	56165021	56280028	2	rs436362295^a^	56165021	C	0.016	2.18 x10^-6^	5	CD37
18	56551941	56711791	9	rs134804275^c^	56645162	T	0.017	1.23 x10^-7^	10	MED25^†^
19	25866591	26579664	2	rs455574605^a^	26440513	A	0.125	1.41 x10^-6^	5	WSCD1
19	28459429	28642278	3	rs378930477^d^	28461407	T	0.103	5.12 x10^-6^	9	CTC1^†^,PFAS,
21	40864588	41148186	23	rs209001772^a^	40955154	T	0.474	1.76 x10^-6^	1	ENSBTAG00000027863
22	49840740	49982657	13	rs379946352^d^	49940652	T	0.974	1.26 x10^-7^	3	DCAF1,RBM15B^†^,DOCK3
26	20495664	20642095	5	rs382274415^a^	20604629	A	0.949	4.51 x10^-6^	5	ABCC2
28	12400473	12409730	5	rs378225261^a^	12401160	C	0.910	7.44 x10^-6^	0	
28	15704269	15727249	2	rs444722974^a^	15704269	C	0.005	2.38 x10^-7^	0	

*BTA, Bos taurus autosome number; SNP, name of single nucleotide polymorphism. No SNP <10^-5^ is the number of SNPs within the QTL with a p-value ≤ 1 × 10^-5^. P-value is the p-value of strongest SNP association. + Allele is the allele that had a positive SNP effect on gestation length. No of genes is the number of genes in the QTL. If no gene was present in the QTL the nearest functional candidate gene within 250 kb was chosen. ^a^intergenic, ^b^upstream gene variant, ^c^intronic, ^d^downstream gene variant,^†^gene in which the most significant SNP within the QTL was identified in.

The QTL with the strongest association in the across-breed analyses was on BTA7 spanning from 52.92 to 53.09 Mb where the intronic variant rs38248541 within *CYSTM1*, which was also significantly associated with gestation length in the HF population, explained 0.75% of the genetic variance in gestation length (**Table 5**). Similarly, the QTL on BTA14 from 44.49 to 48.59 Mb also overlapped a QTL identified in the HF population, although the strongest variant within this QTL in the across-breed analyses was marginally more significant (P = 3.36 × 10^-7^) than that identified in the HF population. Interestingly, the QTL on BTA4 located between 94.32 and 94.82 Mb was not previously identified in any of the within-breed analyses although each of the three suggestively-associated variants were only marginally segregating across all breeds (mean MAF = 0.005). Both the upstream and downstream variant classes were substantially enriched for association across all breeds with fold enrichments of 1.23 and 1.18 detected, respectively.

**Figure 2:**
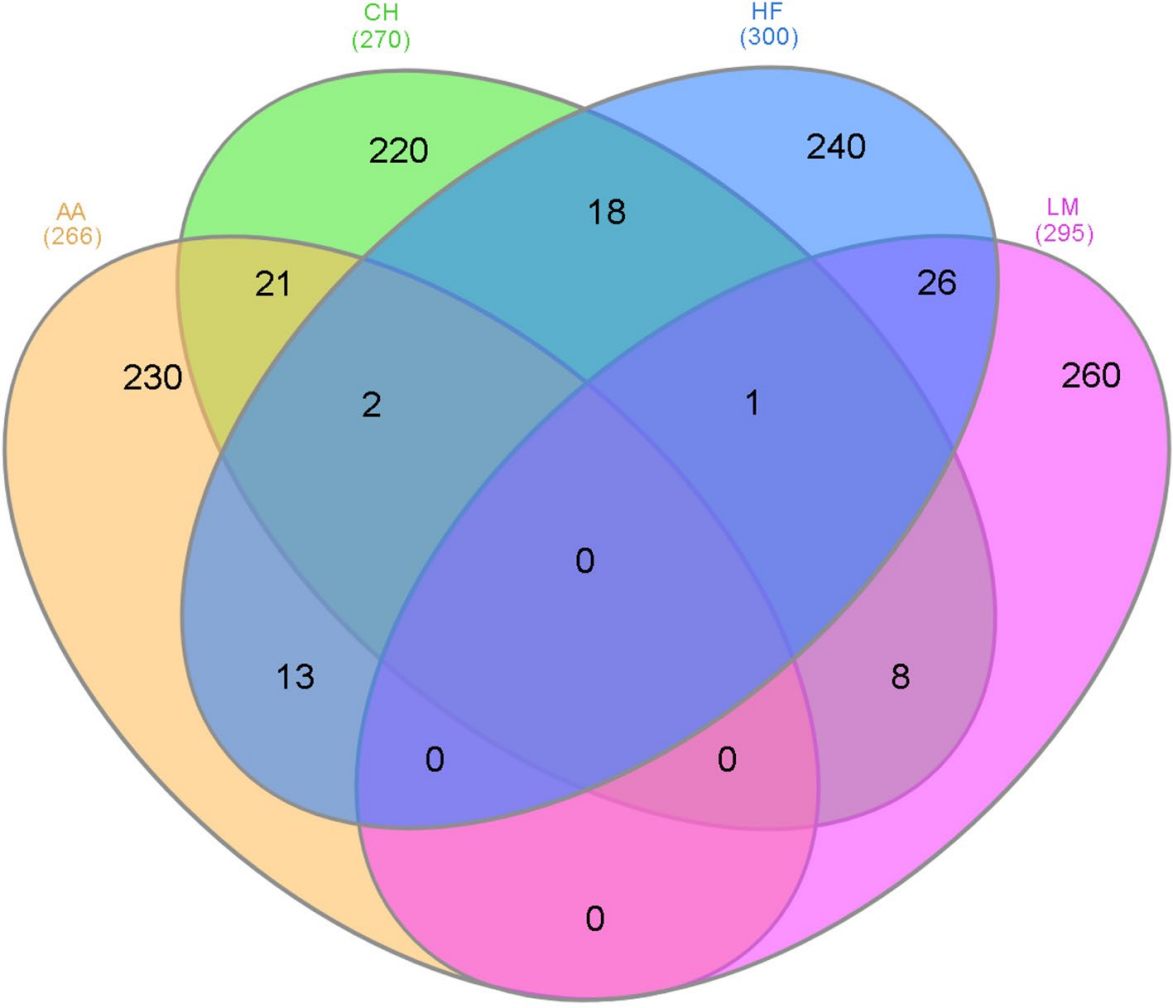
The number of 500 kb windows that contained a single nucleotide polymorphism (SNP) with a p-value ≤1 × 10^-4^ that overlapped in the different breeds where AA represents Angus, CH is Charolais, HF is Holstein-Friesian, and LM is Limousin and the number in parenthesis is the number of 500 kb windows that contained at least one SNP with a p-value ≤1 × 10^-4^.

As genomic regions rather than individual SNPs may be influencing gestation length across all breeds, overlapping 500 kb windows that contained at least one SNP with a P <1 × 10^-4^ within each breed were identified (**Figure 2**). The majority of the 500 kb windows harboring a significant SNP (P < 1 × 10^-4^) were unique to a single breed and no window was significant in all four breeds for gestation length. The greatest number of overlapping windows was between the HF and LM populations where 26 windows across 15 different BTAs were associated with gestation length in both breeds. Two windows were shared across all of the AA, CH, and HF populations whereas one window was common between the CH, HF, and LM populations. Several candidate genes were identified in these windows including *FOXD*, *CMPK1* and *EXOC4* between the AA, CH, and HF populations and *CHRM2* in the CH, HF, and LM populations.

### SNP Effect Directions

**Figure 3:**
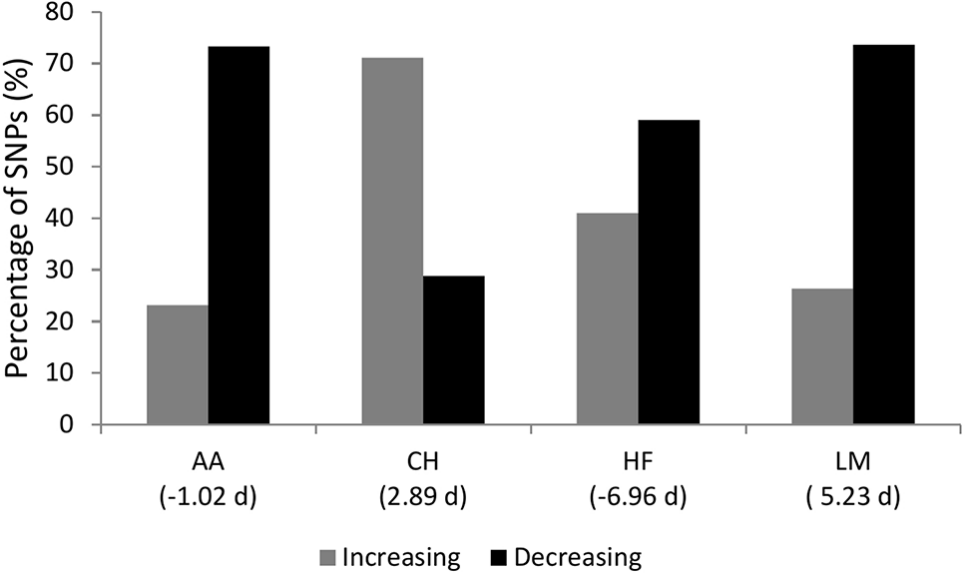
The percentage of single nucleotide polymorphisms (SNPs) with a p-value ≤ 1 × 10^-4^ for gestation length where the major allele SNP effect direction was associated with an increase or decrease in gestation length. AA represents Angus, CH is Charolais, HF is Holstein-Friesian and LM is Limousin. The mean gestation length EBV for each breed in days is in parenthesis.

**Figure 4:**
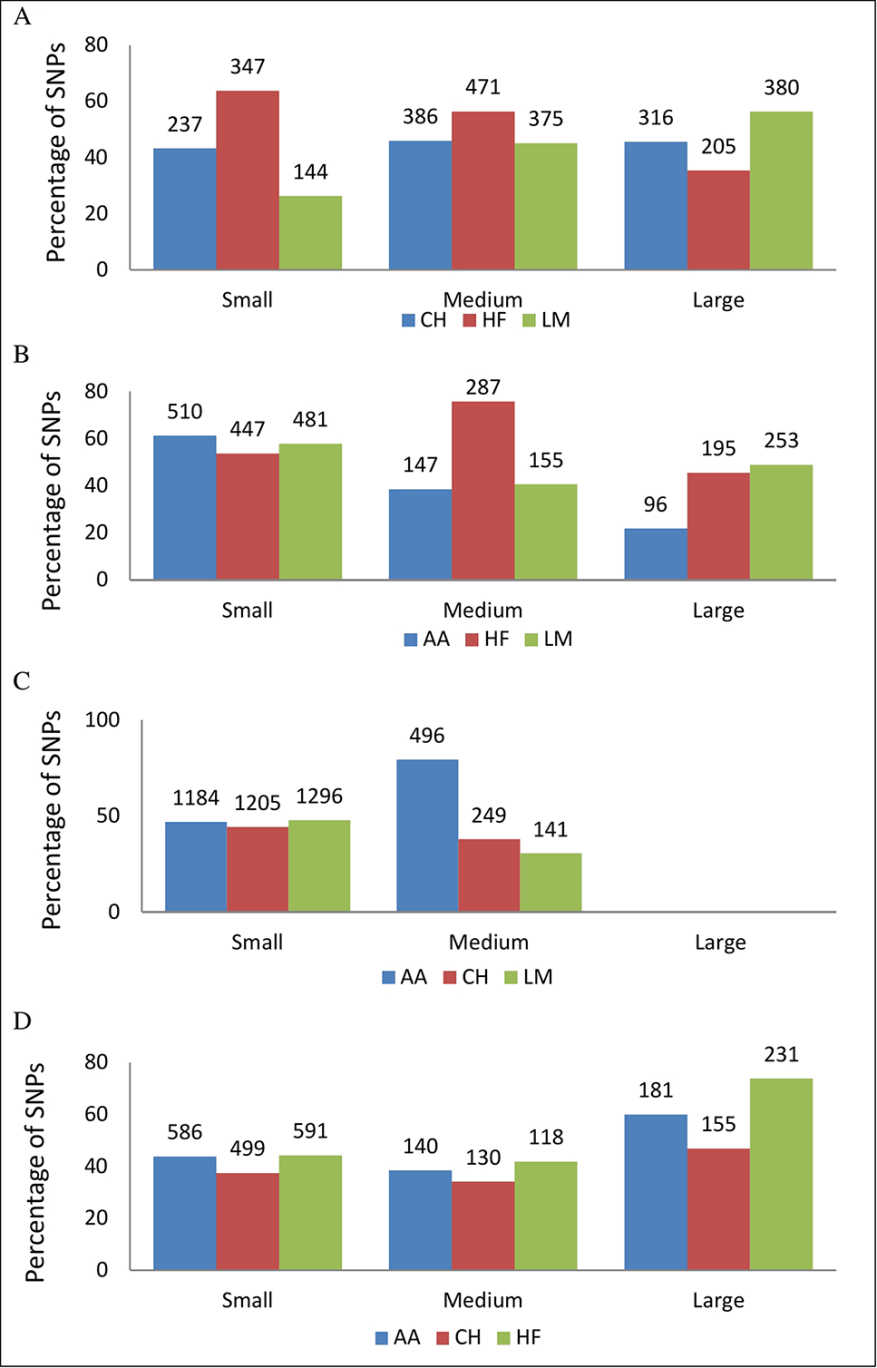
The percentage of single nucleotide polymorphisms (SNPs) in each breed with a p-value ≤ 1 × 10^-4^ for gestation length but with a SNP effect differing between that breed and one of the three breeds it was compared with. SNP effect sizes were categorized into small (-0.8 to 0.8 days), medium (-1.58 to -0.8 and 0.8 to 1.58 days) and large effect sizes (-1.58 or 1.58 days) with the breed of interest and did not have to be consistent when comparing their allele effect direction across breed. **(A)** is Angus, **(B)** Charolais, **(C)** Holstein-Friesian, and **(D)** Limousin. The number of SNPs that had a different SNP direction within each breed comparison is above the bar.

The sign of the allele substitution effect for the major allele in the majority of SNPs with a P ≤ 1 × 10^-4^ was associated with reducing gestation length in all but the CH population where the major allele was associated with a shorter gestation length in only 28.84% of SNPs (**Figure 3**). Irrespective of the allele substitution effect size, the sign of the allele substitution effects were not consistent across breeds (**Figure 4**). Within the HF population, as only one SNP was classified as having a large effect size it was not included in the comparison for the large effect size category within **Figure 4**. Indeed, 77.58% of SNPs were classified into the small effect category (i.e., a SNP effect size between -0.8 and 0.8 days) within the HF population. The inconsistency in the sign of the allele substitution effect for the large effect size category was greatest between the LM and HF populations, where 73.80% of the large effect SNPs that were segregating within the LM population differed in their effect direction to that in the HF population.

### Over-Represented GO Terms and Pathways

There was no significantly over-represented GO term associated with gestation length in any of the populations after adjustment for multiple testing, although 12 GO terms did have a P-value < 0.05 (**Table S2**). The GO term GO:0007156 which represents the attachment of a plasma membrane adhesion molecule in one cell to an identical molecule in an adjacent cell was the most significant of all terms (P = 7.6 × 10^-5^) and a total 10 genes were represented by this term in the HF population. Similarly, there was also no significantly-associated KEGG pathway identified after adjustment for multiple testing but five were identified (P < 0.05) in the HF population and one each in the CH and LM populations, respectively (**Table S2**).

## Discussion

Gestation length, defined as the period between conception and parturition, is often subjected to genetic selection in the pursuit of retaining a compact calving season in seasonal production systems (Vieira-Neto et al., 2017); short gestation length bulls are often used on females served towards the end of the breeding season. From a management perspective, knowledge of the expected calving date (i.e., date of conception plus gestation length) is of huge importance so that nutritional, health and dry-off date decisions can be made at the appropriate time point to ensure optimum cow welfare but also uncompromised performance post-calving. Inter-breed differences in mean gestation length exist (Norman et al., 2009; Fitzgerald et al., 2015); the inter-breed gestation length in Ireland varies from 280.81 days in the HF population to 283.97 days in Herefords (Fitzgerald et al., 2015). Whilst previously documented QTL associated with gestation length (Maltecca et al., 2011; Fang et al., 2019) have been reaffirmed in the HF population used in the present study, many of the QTLs identified in the beef breeds herein were novel but also unique to each of the breeds investigated. This is the first study to identify gestation length QTLs in beef breeds using imputed whole genome sequence, but it is also the first to compare these regions in both dairy and beef breeds.

Although several factors such as the sex of the calf, dam parity, season of conception and whether the animal was a twin or not have all been demonstrated to associate with gestation length (Norman et al., 2009; Fitzgerald et al., 2015), the moderate heritability and documented genetic variation (Hansen et al., 2004; Mujibi and Crews, 2009; Norman et al., 2009) suggests that changing gestation length is indeed possible through genetic selection. A gestation length of between 274 and 281 days has been reported to be optimal for maximizing lifetime productivity, calving ease, perinatal mortality, culling and dam fertility within a US Holstein population (Norman et al., 2009). Hence, based on the current gestation length in Ireland (Fitzgerald et al., 2015), considerable scope for shortening gestation length (up to one week) exists; little is known about the optimal gestation length in beef cattle. Overall, the major alleles at all SNPs with a P ≤ 1 × 10^-4^ in the present study were predominantly associated with a shortening in gestation length, with the exception of the CH population. This suggests that selection for shorter gestation length would be most beneficial in the CH population, where a mean gestation length EBV of 2.89 days among CH sires was reported (**Figure 3**). It is also important to take cognizance, however, that too short a gestation length is also associated with a greater probability of perinatal mortality owing to the non-linear relationship between these traits (Johanson and Berger, 2003). Notwithstanding this, as with any trait, cognizance of other traits needs to be considered when attempting to alter gestation length through breeding. Therefore, to aid in the genetic improvement of gestation length, breeding programs need to facilitate a balanced selection approach with other correlated traits also included in the breeding objective as exists for most populations (e.g., Miglior et al., 2005; Roche et al., 2018; Purfield et al., 2019).

The known association between gestation length and all of calving ease, perinatal mortality, birth weight and fertility (Hansen et al., 2004; VanRaden et al., 2004; Mujibi and Crews, 2009; Norman et al., 2009) is reflected in the present study by the overlap between the detected QTLs for gestation length in the present study with QTLs reported for these correlated traits in the CattleQTL database (Accessed; 1 May 2019); between 5.88% in the CH population and 50.00% of the QTLs associated with gestation length in the across-breed analysis in the present study overlapped with previously identified calving, birth weight and fertility performance QTLs. Moreover, multiple QTLs reported for gestation length in the CattleQTL database were reaffirmed in the present study within the HF and CH population although no overlap was evident for the QTLs reported in the AA, LM and the across-breed analysis. These confirmed QTL, all of which harboured SNPs previously associated with gestation length in both US Holstein and Italian Brown Swiss populations (Maltecca et al., 2011), included the QTL from 48.56 to 48.63 on BTA7 in the CH population, and four QTLs in the HF population (from 51.61 to 56.918 on BTA7, from 57.48 to 57.59 on BTA18, from 58.06 to 58.19 on BTA18 and from 32.88 to 38.47 on BTA19). In total, 75 of 88 SNPs identified by Maltecca et al. (2011) were segregating with a MAF > 0.005 in our HF population and 15 had a P-value < 0.05.

### Major QTL for Gestation Length on BTA 18

The strongest association with gestation length detected was located on BTA18 in the HF population. BTA18 has long been associated with both fertility and calving related traits in cattle, with several studies reporting a QTL around the 55 to 58 Mb position that had a strong association with reproduction outcome (Gaddis et al., 2016), calving ease (Cole et al., 2009; Purfield et al., 2014; Purfield et al., 2015), birth-weight (Cole et al., 2014), or gestation length (Maltecca et al., 2011; Fang et al., 2019) in Holsteins. This was also evident at the level of the individual SNPs, in that the strongest SNP association identified in the present study, rs381577268, the downstream variant of *ZNF613*, was also the strongest SNP association in a recent gestation length association study completed by Fang et al. (2019) based on 27,124 Holstein Bulls with circa. 3 million sequence variants. To confirm if this downstream SNP also had a significant association with direct calving difficulty in our HF population, rs381577268 was tested in a single SNP regression and was found to be highly significant (p = 1.63 × 10^-9^). A similar finding was also reported by Fang et al. (2019) where *ZNF613* was identified as the candidate gene for three additional traits; sire calving ease, body depth, and conception rate, all of which are strongly correlated with gestation length (Hansen et al., 2004; Norman et al., 2009). Although multiple candidate genes were located within the two neighboring QTLs on BTA 18 (57.48 to 57.59 Mb and 58.05 to 58.19 Mb) in the present study, Fang et al. (2019) demonstrated strong supporting evidence that it was the difference in the methylation patterns of the second intron of *ZNF613* that was likely causing a lengthening of gestation and subsequently a difficult calving, a bigger body size but a superior conception rate. In total, 65 SNPs within *ZNF613* were segregating in our HF population including 1 missense variant out of a potential 18 SNPs, but only 2 intronic variants, rs381761581 and rs38460285, were suggestively associated with gestation length and these were located in the third and fourth introns, respectively. None of the 10 segregating SNPs in the second intron were associated with gestation length in the present study but, as it was the methylation pattern rather than the DNA variation within this site that was lengthening the gestation, this was not unexpected. The identification of the same SNP as the strongest association within two separate HF populations (albeit likely related given the relatively low effective population size in HF; Mc Parland et al., 2007), strengthens the argument for *ZNF613* as a likely candidate gene for this QTL region. However, the targeted selection of this candidate gene within breeding programs is likely to have little impact in genetic improvement for shorter gestations as the C allele, which was associated with a shorter gestation length, was near fixation (allele frequency of 0.980) within the HF population; a similar frequency of 0.93 in the U.S Holstein population was reported by Fang et al. (2019). The near fixation of this allele for shorter gestation in the population of HF bulls extensively used in Ireland is not overly surprising giving the reliance on compact seasonal calving in Ireland (Berry et al., 2013) and the relatively strong selection pressure placed in Irish dairy breeding programs on both calving interval (of which maternal gestation length is a component) and direct gestation length.

As the present study was uniquely positioned to identify across-breed QTLs, SNPs within and up/downstream of *ZNF613* were also evaluated for an association with gestation length in any of the three beef breeds in the present study. In total, between 108 and 112 SNPs were segregating within or 2 kb up/downstream of *ZNF613* in each of the beef breeds, but no SNP was even suggestively associated with gestation length, suggesting this QTL on BTA18 is specific to HF. Nevertheless, the low significance (0.001 < P < 0.05) of a genomic region 40 kb downstream of *ZNF613* in both CH and LM does suggest that perhaps if the beef sample population size was larger, a suggestive QTL may have been detected. As this lowly significant region within the CH and LM populations was further upstream than *ZNF613*, there is the suggestion that this QTL may influence gestation length using a different mechanism in the beef population to what was proposed in the HF population (Fang et al., 2019).

### Additional Dairy QTL

Fang et al. (2019) reported 85 SNPs to be significantly associated with gestation length in a US Holstein population, of which 58 were segregating with a MAF >0.005 in our HF population and, of these, 49 SNPs had a P < 0.05. These 49 SNPs were located on BTAs 5, 7, 10, 14, 18, 19, 28 and resided within four of the QTLs identified in the present study, including the QTL on BTA18 as discussed previously. The first of the three remaining QTLs was located on BTA7 in the QTL extending from 51.61 to 56.33 Mb where the most significant SNP in the present study was an intronic variant within *CYSTM1*; *CYSTM1* was also proposed as a candidate gene for gestation length by Fang et al. (2019) in US dairy cows and by Meszaros et al. (2018) in an Austrian and German Fleckvieh dairy cow population. Although the role of *CYSTM1* in gestation length is not clear, it has been shown to play a role in the response to stress or pathogens (Venancio and Aravind, 2010) and has been previously associated with body mass index in humans (Winkler et al., 2015).

Sixteen genes were located in the QTL on BTA10 from 42.27 to 43.30 Mb but only one of the 21 suggestively-associated SNPs was not an intergenic variant and it was located within *CDKL1*; *CDKL1* was also proposed as a candidate gene by Fang et al. (2019) in their study of gestation length in cattle and is essential for meeting the high demand of purines for nucleic acid synthesis in early embryonic development (Michot et al., 2017). The last of the overlapping QTL from the present study and that of Fang et al. (2019) was the QTL from 28.45 to 28.98 Mb on BTA19 which harboured 15 genes (**Table 4**); both MYH10 and NDEL1 within this QTL were selected as putative candidate genes by Fang et al. (2019). However, since our strongest association was further upstream than both *MYH10* and *NDEL1*, we propose *PFAS* as the most likely candidate gene within our population. A missense mutation within *PFAS* has been previously identified as the likely cause of the embryonic lethal MH1 haplotype in the Montbeliarde breed and homozygous carriers of this mutation typically died between 7 and 35 days post insemination (Michot et al., 2017). The intronic SNP rs378360129 within the *PFAS* gene was the second strongest association within this QTL but the causative missense mutation, rs455876205, as proposed by Michot et al. (2017), was not segregating in our HF population. Indeed no significant segregating missense mutation within *PFAS* was identified, although not all of the missense variants within *PFAS* are likely to have been captured during sequencing, and perhaps *PFAS* could be affecting gestation length through a different mechanism within our population. Lastly, although not identified as a putative QTL in the present study, 31 of the 49 genome-wide significant SNPs identified by Fang et al. (2019) located between 1.31 and 2.09 Mb on BTA14, where marginally significant in our population with p-values ranging from 1 × 10^-4^ to 0.05. Perhaps if our population size was comparable in size to Fang et al. (2019) which was based on 27,124 animals, this QTL region would have reached significance within our HF population.

### QTLs for Gestation Length in Beef

Although multiple putative QTL regions within the AA, CH and LM populations were identified in the present study, little overlap between these and previously identified QTL was evident. This is not unexpected as, to the best of our knowledge, this is the first study that undertook genome-wide associations for gestation length in beef cattle. Nonetheless, one QTL region from 48.56 to 48.63 Mb on BTA7 in the CH population did overlap with a QTL previously documented by Maltecca et al. (2011) in both HF and Brown Swiss populations. Only the *Neurog1* gene was present within this QTL which has been shown to promote neuronal differentiation mid-gestation and was gradually suppressed in late gestation (Sun et al., 2001). One QTL which could be exploited in the CH population to shorten gestation length is that on BTA12 from 66.45 to 66.65 Mb containing the *GPC5* gene. Glypican-5 (*GPC5*) has been shown to be mainly expressed in fetal tissues and is believed to play an important role in growth and differentiation during development through regulation of Wnt, hedgehog, fibroblast growth factor and bone morphogenetic protein pathways in humans (Saunders et al., 1997; Li et al., 2011). The major allele at all suggestively-associated SNPs within *GPC5*, although near fixation, was associated with longer gestation. This suggests that if the allele frequency of the minor alleles could be increased within this QTL, a potential shortening in the gestation length within the CH population could be achieved. Additional potential candidate genes include *ASB5* and *MYCBP2* identified in the AA and LM populations, respectively. Although the QTL surrounding the *ASB5* gene on BTA27 in the AA population was the strongest association within this breed, the role of ASB5 in gestation length is unclear. It has, however, been shown to be expressed in skeletal muscle and is believed to play a role in myogenic satellite cell lineage (Tee and Peppelenbosch, 2010) which suggests it may play a developmental role during gestation. Interestingly, *MYCBP2* which was identified as a candidate gene for gestation length in the LM population was previously proposed as a candidate gene for preeclampsia within European humans (Zhao et al., 2013).

### Across-Breed QTLs

Limited across-breed QTLs were identified in the present study and those that were identified from the across-breed association analysis of all 22,566 sires, were predominantly reflective of those identified in the HF population. Despite this, putative across-breed candidate genes such as *EXOC4* and *FOXD2*, were identified through the overlapping 500 kb window approach. *EXOC4* was identified as a putative across-breed candidate gene between AA, CH and HF populations and has been shown to be regulated in trophoblast differentiation in humans (Gonzalez et al., 2014). In addition, homozygous mice embryos have been shown to die prematurely due to a defect in mesoderm formulation and its absence has been shown to induce genomic instability (Martin-Urdiroz et al., 2016). *FOXD2*, also identified as a putative across-breed candidate gene between AA, CH and HF populations and has been shown to have multiple roles in embryogenesis including mediating the response of cells to signaling molecules such as SHH (Wu et al., 1998).

### Implications and Application

Because of the moderate heritability of gestation length cattle (0.33 to 0.62; Hansen et al., 2004; Mujibi and Crews. 2009; Norman et al., 2009), the true breeding value of an animal for gestation length should correlate strongly with its phenotypic value. Hence, being able to predict the breeding value of an animal can be informative in predicting the phenotype. The maximum accuracy with which the true breeding value of an animal can be predicted, however, from solely pedigree information is 0.70; theoretically, the accuracy can be one if genomic information is used. From an analysis of >1 million records from US dairy cows, Norman et al. (2009) reported a difference of at least 10 days in predicted transmitting ability for direct gestation length among sires. Given that the sire only accounts for one-quarter of the genetic variance, the extent of genetic variability in the resulting progeny could therefore be multiples of this. Genotyping of embryos for at least circa 50,000 genetic markers is now possible (Le Bourhis et al., 2011) and thus genomic prediction of gestation length of individual embryos as a management aid for predicting calving date is now possible. Nonetheless, once a phenotype for gestation length of an animal already exists, then the marginal benefit of genomic prediction is expected to be small given the moderate heritability; assuming a heritability of 0.48 (i.e., mid-point of 0.33 to 0.62; Hansen et al., 2004; Mujibi and Crews. 2009; Norman et al., 2009) then the accuracy of selection based on just the animal’s own record (i.e., ignoring parental contribution) is 0.69.

Genomic predictions for many traits, irrespective of how complex the phenotype, are generally presented as just a single value such as a predicted 1-day shorter gestation length. Having knowledge of the underlying genetic variants contributing to these genomic predictions, even if not causal, has potential use in sire mating advice decision support tools (Carthy et al., 2019). For example, as previously alluded to, the C allele of the rs381577268 SNP downstream from the *ZNF613* gene which was associated with shorter gestation in the HF was almost fixed. Because of this, a candidate sire of sires and dam of sires could have a genomic prediction for shorter gestation (although it is not guaranteed given the polygenic nature of the trait; **Figure 1**), but, because the allele is almost fixed, there is no further genetic gain to be achieved at this locus. Moreover, if both parents are homozygous for a segregating allele that confers a shorter gestation, then the expected genomic merit of the resulting progeny can be predicted with greater accuracy (i.e., it has to be homozygous). This could be particularly important in the pursuit of a trait with an intermediate optimum, like gestation length.

Detection of alleles associated with gestation length in multiple breeds can be useful in a) retaining a high accuracy of genomic predictions over generations, and b) exploiting phenotypic and genomic information from one breed to augment the accuracy of genomic predictions in another breed. The issue of eroding genomic predictions over generations should, however, be less of an issue in a well-structured breeding program coupled with a well-designed and operated phenotyping strategy as the genomic predictions are constantly being updated with every new generation thereby negating the impact of breakdown in linkage disequilibrium over time; maintaining accurate genomic predictions over time is more of an issue in populations with a less-developed breeding and phenotyping strategy or for phenotypes that are resource-intensive to measure such as feed intake. Results from the present study though, which is in general agreement with many other studies (Purfield et al., 2019; Doyle et al., 2019), is that most proposed SNPs or QTLs do not appear to associate with the phenotype of interest in all breeds investigated.

A further use of locating more definitively the region of the genome associated with, or ideally affecting, gestation length is the ability to identify animals that excel in gestation length without compromising performance in correlated traits. Genetic correlations among traits are a manifestation of either pleiotropy or linkage. While pleiotropy cannot be resolved through knowledge of the underlying causal variants, antagonistic correlations among traits can be weakened in a breeding program that uses germplasm where the alleles conferring the antagonistic effects do not appear together. Therefore, it is plausible that the negative correlation between a short gestation length and high calf mortality could be disentangled.

## Conclusions

This is the first study globally in cattle that has attempted to locate genomic regions associated with direct gestation length in both dairy and beef cattle. Even when the genome was split into 0.5 Mb segments, the majority of such segments harboring a significant SNP (P ≤ 1 × 10^-4^) were unique to a single breed with no segment common to all four breeds. This has obvious implications for their usefulness in genomic predictions that can effectively operate across multiple different breeds, at least for the breeds evaluated in the present study.

## Data Availability Statement

Sequence variant genotypes were provided by participation in the 1000 Bulls consortium and can be found at NCBI BioProject PRJNA238491, PRJEB9343, PRJNA176557, PRJEB18113, PRNJA343262, PRJNA324822, PRJNA324270, PRJNA277147, PRJNA474946, and PRJEB5462. For the remaining sequences the board of the 1000 Bull Genome Consortium should be contacted. Individual genotype and phenotype data used in this study is also managed by a third party, the Irish Cattle Breeding Federation. Requests for genotype data can be made to the Irish Cattle Breeding Federation, Highfield House, Shinagh, Bandon, Co. Cork, Ireland: email, query@icbf.com; fax: +353 (0)238820229; phone: +353 (0)238820222; website: www.icbf.com. All significant associations identified in the present study are provided within the manuscript and through additional material.

## Ethics Statement

Ethical review and approval was not required for the animal study because the data used in the present study originated from a pre-existing database managed by the Irish Cattle Breeding Federation (ICBF). Therefore, it was not necessary to obtain animal care and use committee approval in advance of conducting this study.

## Author Contributions

DP and DB participated in the design of the study. DP imputed, analysed the sequence data and drafted the manuscript. RE estimated the breeding values. DB, RE and TC helped to draft the manuscript. All authors read, revised and approved the final manuscript.

## Funding

The project leading to these results has received funding from the European Union’s Horizon 2020 research and innovation programme – GenTORE – under grant agreement No 727213. In addition funding from Science Foundation Ireland principal investigator award grant (14/IA/2576) and the centers award Grant 16/RC/3835 (VistaMilk) is also gratefully appreciated.

## Conflict of Interest

The authors declare that the research was conducted in the absence of any commercial or financial relationships that could be construed as a potential conflict of interest.
