# Supplementary material for: Genomic Regions Associated With Gestation Length Detected Using Whole-Genome Sequence Data Differ Between Dairy and Beef Cattle
Source: Front Genet. 2019 Nov 5;10:1068. doi: 10.3389/fgene.2019.01068 (PMC6848454; doi:10.3389/fgene.2019.01068)
Supplement: Supplementary file 1 [file Table_1.docx]

Supplementary

Supplementary Table 1. The mean estimated breeding values (EBVs) for gestation length and standard deviation (SD) per breed for all purebred sires used in the study and the phenotypic gestation lengths for all purebred females per breed

| Population | Breed | Animal number | Mean EBV | S.D EBV |
| --- | --- | --- | --- | --- |
| Sires | Angus | 2,308 | -1.02 | 3.07 |
|  | Charolais | 2,327 | 2.89 | 2.31 |
|  | Holstein-Friesian | 14,759 | -6.96 | 2.43 |
|  | Limousin | 3,172 | 5.23 | 2.17 |
| Female phenotypic | Angus | 9,640 | 283.28 | 5.99 |
|  | Charolais | 8,511 | 290.21 | 5.61 |
|  | Holstein-Friesian | 1,235,090 | 280.42 | 5.40 |
|  | Limousin | 14,316 | 291.31 | 5.39 |
