# Supplementary material for: Genomic Regions Associated With Gestation Length Detected Using Whole-Genome Sequence Data Differ Between Dairy and Beef Cattle
Source: Front Genet. 2019 Nov 5;10:1068. doi: 10.3389/fgene.2019.01068 (PMC6848454; doi:10.3389/fgene.2019.01068)
Supplement: Supplementary file 2 [file Table_2.docx]

Supplementary Table 2. Over represented GO terms and pathways (P <0.05) associated gestation length across four breeds*

| Analysis | Breed* | Term | Description | P-value | Genes |
| --- | --- | --- | --- | --- | --- |
| Go Terms | |  |  |  |  |
|  | AA | GO:0007156 | homophilic cell adhesion via plasma membrane adhesion molecules | 0.029 | ENSBTAG00000019876, ENSBTAG00000015238, ENSBTAG00000040584 |
|  | CH | GO:0007204 | positive regulation of cytosolic calcium ion concentration | 0.002 | ENSBTAG00000007159, ENSBTAG00000012328, ENSBTAG00000020467 |
|  |  | GO:0009755 | hormone-mediated signaling pathway | 0.021 | ENSBTAG00000012328, ENSBTAG00000017489 |
|  |  | GO:0048146 | positive regulation of fibroblast proliferation | 0.043 | ENSBTAG00000007159, ENSBTAG00000008409 |
|  | HF | GO:0007156 | homophilic cell adhesion via plasma membrane adhesion molecules | 7.69x10^-5^ | ENSBTAG00000007349, ENSBTAG00000030227, ENSBTAG00000012518, ENSBTAG00000037885, ENSBTAG00000039162, ENSBTAG00000030474, ENSBTAG00000039433, ENSBTAG00000008437, ENSBTAG00000046717, ENSBTAG00000017349 |
|  |  | GO:0019372 | lipoxygenase pathway | 0.006 | ENSBTAG00000011990, ENSBTAG00000031933, ENSBTAG00000021933 |
|  |  | GO:0045766 | positive regulation of angiogenesis | 0.021 | ENSBTAG00000008723, ENSBTAG00000020983, ENSBTAG00000016344, ENSBTAG00000020921, ENSBTAG00000005198, ENSBTAG00000021933 |
|  |  | GO:0019369 | arachidonic acid metabolic process | 0.023 | ENSBTAG00000011990, ENSBTAG00000031933, ENSBTAG00000021933 |
|  |  | GO:2001303 | lipoxin A4 biosynthetic process | 0.036 | ENSBTAG00000011990, ENSBTAG00000021933 |
|  |  | GO:0070373 | negative regulation of ERK1 and ERK2 cascade | 0.037 | ENSBTAG00000008010, ENSBTAG00000008250, ENSBTAG00000005367, ENSBTAG00000017120 |
|  | ALL | GO:0051056 | regulation of small GTPase mediated signal transduction | 0.002 | ENSBTAG00000027151, ENSBTAG00000015805, ENSBTAG00000014178 |
|  | | GO:0006810 | transport | 0.011 | ENSBTAG00000037526, ENSBTAG00000018808, ENSBTAG00000002982, ENSBTAG00000000071 |
| KEGG pathways | | |  |  |  |
|  | CH | bta05206 | MicroRNAs in cancer | 0.009 | ENSBTAG00000029764, ENSBTAG00000008409, ENSBTAG00000029802, ENSBTAG00000029951 |
|  | HF | bta04640 | Hematopoietic cell lineage | 0.002 | ENSBTAG00000011421, ENSBTAG00000015032, ENSBTAG00000004909, ENSBTAG00000016284, ENSBTAG00000038045, ENSBTAG00000019498, ENSBTAG00000004608 |
|  |  | bta00240 | Pyrimidine metabolism | 0.016 | ENSBTAG00000030820, ENSBTAG00000047186, ENSBTAG00000004931, ENSBTAG00000004651, ENSBTAG00000011083, ENSBTAG00000008428 |
|  |  | bta04750 | Inflammatory mediator regulation of TRP channels | 0.020 | ENSBTAG00000005978, ENSBTAG00000010576, ENSBTAG00000020921, ENSBTAG00000031933, ENSBTAG00000021933, ENSBTAG00000003014 |
|  |  | bta04912 | GnRH signaling pathway | 0.041 | ENSBTAG00000021766, ENSBTAG00000013392, ENSBTAG00000001014, ENSBTAG00000010576, ENSBTAG00000038735 |
|  |  | bta04015 | Rap1 signaling pathway | 0.043 | ENSBTAG00000004915, ENSBTAG00000005978, ENSBTAG00000020979, ENSBTAG00000007581, ENSBTAG00000020983, ENSBTAG00000010576, ENSBTAG00000020921, ENSBTAG00000005198 |
|  | LM | bta04530 | Tight junction | 0.003 | ENSBTAG00000026266, ENSBTAG00000010581, ENSBTAG00000016024 |

*AA represents Angus, CH is Charolais, HF is Holstein-Friesian, LM is Limousin and ALL represents all four breeds cumulatively.
